# Can gaze control steering?

**DOI:** 10.1167/jov.23.7.12

**Published:** 2023-07-21

**Authors:** Samuel Tuhkanen, Jami Pekkanen, Callum Mole, Richard M. Wilkie, Otto Lappi

**Affiliations:** 1 Cognitive Science, TRUlab, University of Helsinki, Helsinki, Finland; 2 Cognitive Science, University of Helsinki, Helsinki, Finland; 3 Alan Turing Institute, London, UK; 4 School of Psychology, University of Leeds, Leeds, UK

**Keywords:** eye tracking, steering, guidance, waypoints, visuomotor control

## Abstract

When steering a trajectory, we direct our gaze to locations (1–3 s ahead) that we want to steer through. How and why are these active gaze patterns conducive to successful steering? While various sources of visual information have been identified that could support steering control, the role of stereotypical gaze patterns during steering remains unclear. Here, experimental and computational approaches are combined to investigate a possible direct connection between gaze and steering: Is there enough information in gaze direction that it could be used in isolation to steer through a series of waypoints? For this, we test steering models using waypoints supplied from human gaze data, as well as waypoints specified by optical features of the environment. Steering-by-gaze was modeled using a “pure-pursuit” controller (computing a circular trajectory toward a steering point), or a simple “proportional” controller (yaw-rate set proportional to the visual angle of the steering point). Both controllers produced successful steering when using human gaze data as the input. The models generalized using the same parameters across two scenarios: (a) steering through a slalom of three visible waypoints located within lane boundaries and (b) steering a series of connected S bends comprising visible waypoints without a visible road. While the trajectories on average broadly matched those generated by humans, the differences in individual trajectories were not captured by the models. We suggest that “looking where we are going” provides useful information and that this can often be adequate to guide steering. Capturing variation in human steering responses, however, likely requires more sophisticated models or additional sensory information.

## Introduction

Studies of human steering have demonstrated tight coupling between eye movements and steering behaviors (Land & Lee, 1994; Wilkie & Wann, 2003b; Kountouriotis et al., 2013; Mole, Kountouriotis, Billington, & Wilkie, 2016; Lappi et al., 2020; Tuhkanen, Pekkanen, Wilkie, & Lappi, 2021). Typical gaze behavior during steering is a repeating pattern of eye movements, comprising smooth pursuit tracking of a point on the ground (for approximately 0.5 s) followed by a saccade to a new point at a time headway 1–3 s ahead (Lappi & Lehtonen, 2013; Lappi, Pekkanen, & Itkonen, 2013; Lehtonen, Lappi, Kotkanen, & Summala, 2013; Tuhkanen et al., 2019). This pattern is intermittently broken by forward-polling “look-ahead” fixations further ahead (Lehtonen et al., 2013; Mole, Pekkanen, Sheppard, Markkula, & Wilkie, 2021), return fixations closer to the observer (Navarro et al., 2021), and scanning of the scenery and vehicle instruments (for review, see Lappi, 2022). Such gaze patterns have been observed in other contexts such as walking (Imai, Moore, Raphan, & Cohen, 2001; Matthis, Yates, & Hayhoe, 2018) and cycling (Vansteenkiste, Cardon, D'Hondt, Philippaerts, & Lenoir, 2013) and therefore seem to represent a general, robust visual strategy during locomotion.

In terms of how gaze and steering are functionally related, we know that gaze leads steering by about 1 s (e.g., when steering to the right, the head and eyes will move rightward first, followed by the steering wheel; Chattington, Wilson, Ashford, & Marple-Horvat, 2007). Furthermore, various studies have demonstrated that manipulating gaze behavior can bias steering (Readinger, Chatziastros, Cunningham, Bülthoff, & Cutting, 2002; Wilkie & Wann, 2003b; Robertshaw & Wilkie, 2008; Kountouriotis, Floyd, Gardner, Merat, & Wilkie, 2012). The possible causal mechanisms to explain “how” and “to what extent” gaze informs steering have been more difficult to determine.

For example, sometimes the role of gaze control in supporting skilled human behavior is linked with the need to direct more accurate foveal vision onto successive visual targets. When reading text, for example, a characteristic pattern of eye movements is well explained by the need to sample visual information from the point of fixation (Rayner, 1998); due to crowding and/or lower visual acuity, individual characters in text are difficult to resolve using the visual periphery. In contrast, when steering, useful information is available from the whole of the visual field (Wolfe, Dobres, Rosenholtz, & Reimer, 2017), and it is less clear that there is unique information available directly at the point of fixation. So what is the purpose of fixating (and tracking) specific points in the scene?

Another possibility is that the act of directing gaze toward a point when steering effectively produces a transformation of retinal visual signals that in itself supplies information useful for controlling steering. Such signals would include the pattern of optic flow on the retina (after it has been transformed by gaze rotations), which could directly specify steering demands (Kim & Turvey, 1999; Wann & Swapp, 2000; Matthis, Muller, Bonnen, & Hayhoe, 2022). Another possibility is that (perhaps irrespective of where exactly one is looking) visual input from each fixation can be processed to glean geometrical information from the entire scene layout—for example, the direction of “the tangent point” in the visual field (Land & Lee, 1994) or the directions and (relative) distances of interception targets (waypoints; Wilkie, Wann, & Allison, 2008). Finally, when there is a change in the angle of the head and eye relative to the body, extraretinal gaze direction signals are provided that can be useful for steering (Wilkie & Wann, 2003a, 2005). On the sensory side, these changes can be registered proprioceptively, whereas on the motor side, the “efference copy” of gaze control could provide a useful control signal (Donaldson, 2000).

When fixating waypoints (interception targets on the ground), information related to the direction and distance of each point in the world could thus also be made available in the form of oculomotor output: If the gaze control system “places” the gaze point in the world, then that place can automatically become a target for the steering system to intercept. Of course, some kind of mapping from oculomotor to locomotor coordinates would need to be in place. Perhaps the depth distance to the point of fixation and the horizontal angle of gaze relative to the current direction of travel are used to specify the path curvature needed to intercept the point of fixation, or alternatively, a point of fixation with a 2-s time headway and the gaze angle could be used to specify the required rate of rotation. By either method, the gaze control information could be useful in itself as a (nonvisual) steering input. If such information is available from the oculomotor system, and sufficient to support steering, then, somewhat counterintuitively, looking where we are going might not be so much to see *what* is there (visual analysis of local features at the point of fixation), but rather to tell the rest of the motor system *how* to move there.

### Aims of the study

**Figure 1.**
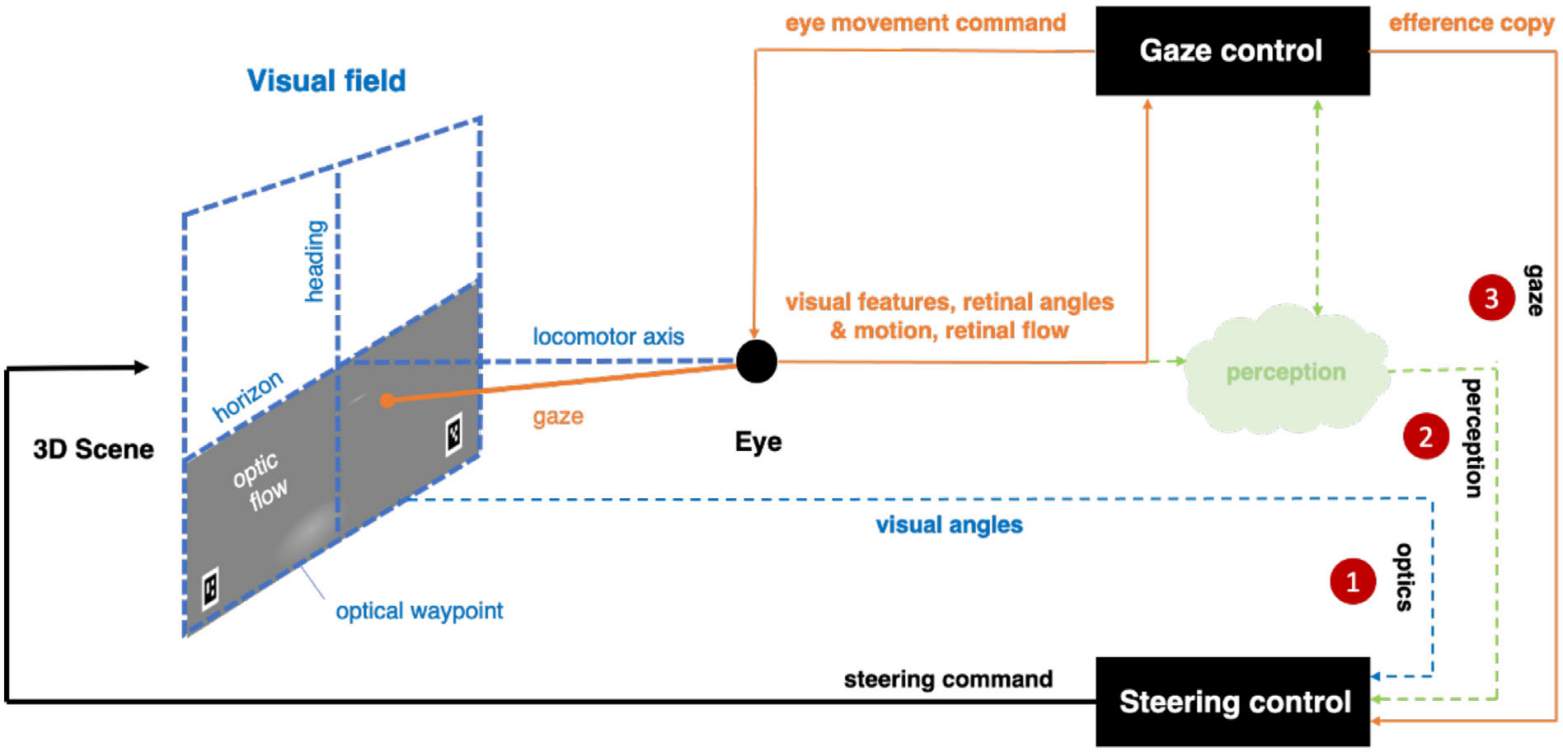
The steering by gaze concept. (1) Traditional steering models steer directly from the optics (i.e., environmental texture projected to the point of vantage as optic flow or the visual angle of a steering point relative to locomotor heading). (2) These stimulus features are not, however, present as such in the proximal input (e.g., retinal angles and retinal flow also depend on the movements of the eye), and perceptual processes are commonly thought to intervene between the distal stimulus and the steering response but rarely specified. Proprioceptive and efference copy information of gaze direction is often thought to feed into perceptual processes to allow recovery of the optical information by subtracting changes in retinal input caused by active movement of the eye. (3) The steering-by-gaze concept suggests a complementary role for efference copy from gaze control: It could be used to drive steering control directly that is, sending to the steering controller motor coordinate information about the current (gaze) target, as opposed to first doing perceptual processing to estimate nonmotor (e.g., optical feature) information about that target.

Our aim was to investigate whether gaze direction information (as opposed to optical information from the scene) would be sufficient for controlling steering (see Figure 1 for a visual conceptualization). To achieve this aim, gaze behavior was recorded using a head-mounted eye tracker, while human participants steered a winding path during two simulated driving experiments (lateral steering control but no longitudinal velocity control). We then used the actual human gaze patterns as the steering input for simple controllers, before finally assessing whether these simulations could produce successful steering from extraretinal gaze direction alone.

To determine whether the fit to human steering behavior was specific to the control law being used, two different controllers modeled steering-by-gaze: (a) a proportional controller that sets a target yaw-rate in proportion to the visual angle between gaze/target and locomotor heading and (b) a pure-pursuit controller (Coulter, 1992; Samuel, Hussein, & Mohamad, 2016) that steers on a circular trajectory toward a given steering point in the world, a point of fixation projected on the virtual plane of travel. The actual gaze data of participants were used to derive the steering points of the controllers.

The aim was to take the simplest possible controller that we could imagine (the proportional controller) as well as a more commonly used but still simple controller (the pure-pursuit controller) to examine how sensitive steering by gaze control was to the specifics of the control law. In addition to testing steering-by-gaze, we also reran each model using optically available waypoints as steering points (instead of using gaze) to establish how well the given controllers could in principle steer when using the available optical information.

We ran the models on two different datasets (collected during different steering tasks and with gaze data from different participants in different laboratories) to assess how well the steering models generalized to the second dataset with model parameters that were estimated from the first dataset. The goal was to see how well (if at all) the control laws could operate when human gaze direction was the sole input.

## Methods

### Experimental procedure

**Figure 2.**
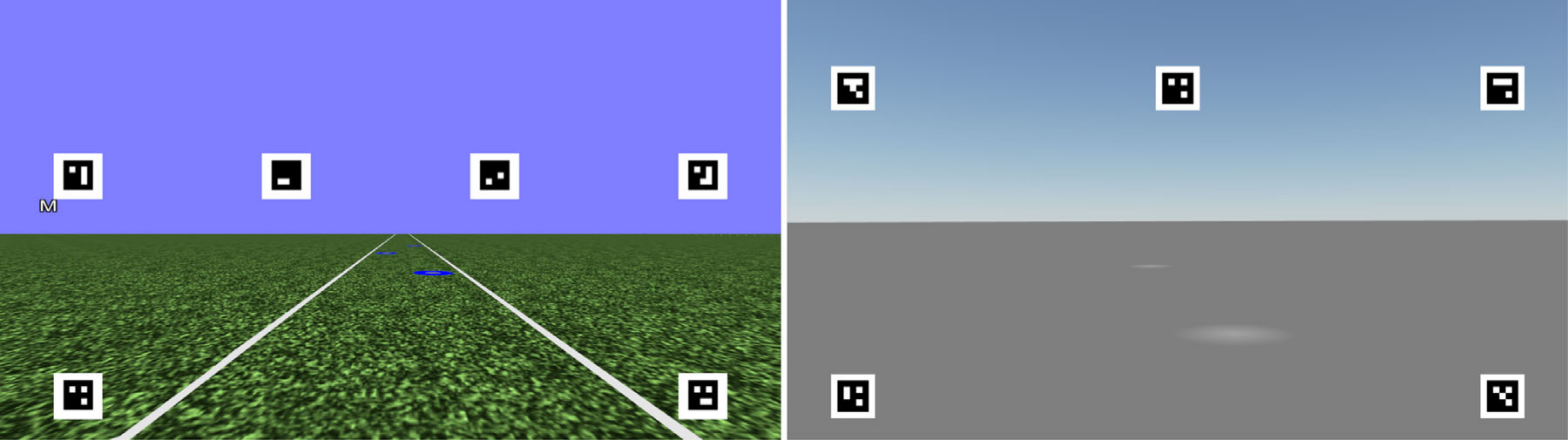
*Left panel.* Sample screenshot from a trial in Experiment 1. The participants were instructed to steer through the blue waypoints. Speed of the virtual vehicle was set constant to 8 m/s. The square optical markers were used to estimate a homography between each frame of the camera and the display in order to determine where the participant gaze was on the display. *Right panel.* Sample screenshot from a trial in Experiment 2. The participants were instructed to steer through a track that was indicated visually by the white waypoints. If the participants veered off the (invisible) 3.5-m-width track, loudspeakers played a beeping sound. Speed of the virtual vehicle was set to (approximately) constant 10.5 m/s.

Two datasets were taken from two separate driving simulator experiments with human participants, where steering and eye movements were recorded (See Figure 2 for sample screenshots). These data were used to simulate steering-by-gaze behaviors (components of these datasets were first presented in Mole et al. [2021] and Tuhkanen et al. [2021]). The two experimental settings and their designs are described briefly below. More detailed descriptions of the equipment and procedures can be found in two previously published studies. Experiment 1 data were collected at the University of Leeds with the same experimental procedure as described in Mole et al. (2021), but in the present article, only the “slalom” sections of each trial were used (the empty straight and bending portions of each trial were not included). Experiment 2 data were collected at the University of Helsinki using the same experimental procedure as described in Tuhkanen et al. (2021), but only the “control trials” were used (the trials with the “missing waypoints” manipulation were not included in the present analysis).

The datasets and code for model implementation/analysis are available at https://github.com/samtuhka/Gaze_Steering_Models.

### Ethics

Data collection for Experiment 1 was conducted at the School of Psychology at the University of Leeds. Participants were briefed on the experiment procedure on arrival and signed an informed consent form for the publication and use of collected data for scientific purposes. The study was approved by the University of Leeds Research Ethics Committee (Ref: PSC435) and complied with the guidelines set out in the Declaration of Helsinki.

The data collection for Experiment 2 was conducted at TRUlab at the University of Helsinki. The participants were debriefed on the experiment procedure and signed an informed consent form for the publication and use of the collected data for scientific purposes. The study was approved by the ethical review board of the University of Helsinki (Ref: Ethical Review Board in the Humanities and Social and Behavioural Sciences, 2017/7) and complied with the guidelines set out in the Declaration of Helsinki and guidelines of the Finnish committee for research ethics (www.tenk.fi).

### Experiment 1

**Figure 3.**
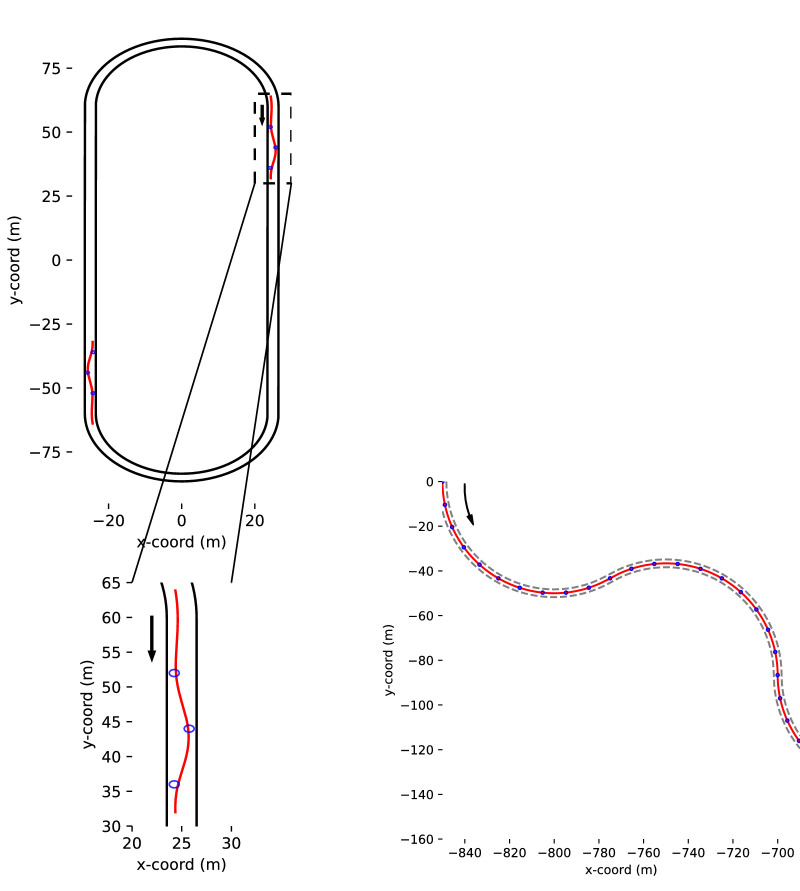
*Left panel.* The complete track in Experiment 1 with a zoomed-in depiction of one of the slalom components below. The slalom sections are depicted in red, and the waypoints the participants were instructed to steer through are shown using blue circles. Direction of travel is clockwise. The red paths are the empirical average “slalom path” as described in the main text. *Right panel.* Section of the Experiment 2 track, consisting of 120-degree arc curves alternating to the left and right. The gray dashed lines indicate the invisible edges, the blue circles the waypoints, and the red line the centerline, which was used as the reference line. The direction of travel is from top-left to bottom-right.

The participants (*N* = 11) steered through a short slalom while also completing a continuous driving task, steering around a larger circuit (see Figure 3). On the straight road section following a right-turning curve, three circular visible waypoints were placed along the road ahead, 8 m from each other (measured along the road centerline), at a 0.75-m horizontal displacement (right, left, and right) from the centerline. The speed of the virtual car was kept constant at 8 m/s. Virtual eye height was 1.2 m above the ground. The simulated environment was displayed at a 60 Hz frame rate and presented on a display with a horizontal field of view of roughly 90°. Note that the vehicle did not have a full physics model. The virtual vehicle moved at the exact set speed, and yaw-rate was in constant proportion to the position of the steering wheel.

The participants were instructed to use the steering wheel to pass over each of the visible waypoints. For the purposes of the analyses, the slalom was determined to start from approximately 12 m before the first waypoint and end 4 m after the last waypoint. The width of the lane was 3 m.

The full dataset contained multiple trial configurations (“narrow” and “wide” separations between waypoints) with the driving sometimes being fully automated, or the participant being tasked to avoid rather than intercept the waypoints. The focus of the current study was on modeling steering-to-intercept behavior, rather than steering-to-avoid (in obstacle avoidance, steering and gaze may be decoupled as drivers may look at the obstacle where they wish *not* to go). Further, the “narrow; intercept” conditions proved to be trivial; drivers could steer over the waypoints with only minor steering adjustments, so gaze and steering coordination appeared relaxed (Vansteenkiste et al., 2013). For the present analysis, therefore, only the “wide; intercept” condition with manual steering control was considered, with the waypoints placed with a 0.75-m displacement from the centerline.

We took the mean trajectory of the human participants as the reference “slalom path” (see the red line in the left panel of Figure 3) and used this to determine how far along the track both the human and model drivers were positioned. For technical purposes, the averaged-out “slalom path” was indexed into discrete positions a few centimeters apart. When the car’s “track position” is referred to later in the article, this means the closest indexed position on the slalom path to the vehicle position (NB: Experiment 2 uses a different reference path). As described in “Model inputs,” the track position was used to derive the gaze input from where the human driver was looking at the corresponding track position. We use the empirical slalom path as the reference line rather than an a priori “optimal path” (such as passing over the centers of the waypoints) because the task of intercepting or “hitting” a waypoint is inherently ambiguous in terms of where the optimal line should lie since it will depend on the characteristic of steering that is being optimized.

### Experiment 2

The participants (*N* = 16) steered through a track consisting of 120-degree arc curves (radius = 50 m) alternating to the left and right (total of 16 curves). The track was visually indicated only by circular white waypoints (see Figure 2), which appeared at a distance of 21 m from the point of observation (along the path centerline, corresponding to a 2-s time headway if driving on the centerline). Waypoints were placed every 10.5 m (i.e., they appeared at an approximate rate of 1/s). The placement of the waypoints in Experiment 2 had been designed to produce gaze behavior akin to that observed in more naturalistic settings, while also minimizing spurious eye movements from non-steering-related tasks. The speed of the virtual car was kept constant at approximately 10.5 m/s. Virtual eye height was at 1.2 m above the ground.

**Figure 4.**
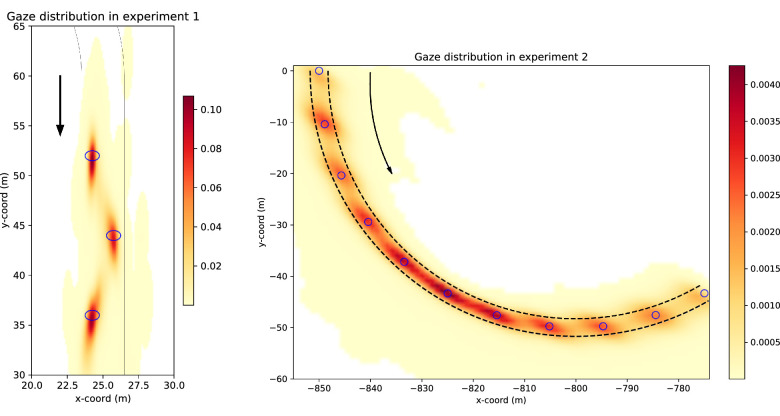
Gaussian kernel-density estimates of points of fixation (locations where the gaze vector intercepts the plane of travel) in Experiments 1 and 2. The color coding indicates the gaze density estimate per square meter (i.e., what proportion of projected gaze points is estimated to fall on each part of the track). *Left panel.* Gaze density distribution in Experiment 1. The right-turning oval track contained two identical slalom sections as depicted in Figure 3 (we treat them as one, identical slalom in our data). The blue circles indicate the location of the waypoints the participants were instructed to steer through. The black lines indicate the visible edge lines. The arrow indicates the direction of travel from top to bottom. *Right panel.* Gaze density distribution in Experiment 2. All right- and left-turning curves have been transformed to matching coordinates (i.e., the depicted curve contains data from all curves). The blue circles indicate the visible waypoints and the dashed black lines the invisible road edge lines. The arrow indicates the direction of travel from top-left to bottom-right.

**Figure 5.**
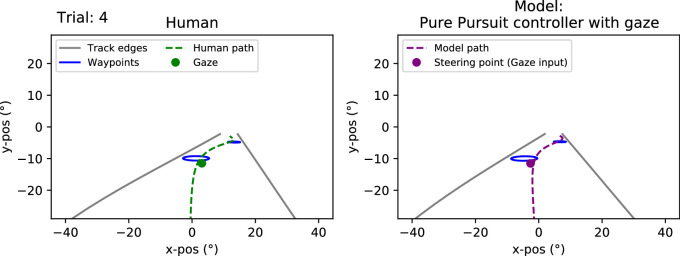
Experiment 1. Sample frame from the Supplementary Movies. The full movies depict the model runs from a first-person perspective side-by-side with the trial from the human driver. *Left panel.* Human driver’s view. The green circle depicts the screen coordinates of the gaze signal as recorded by the eye tracker. The dashed green line depicts the driver’s future path (the actual trajectory they will take). The blue circles depict the visible waypoints and the gray lines the visible track edges. *Right panel.* The model driver’s view in the corresponding trial and track position. The purple circle depicts the steering point, which in this case is based on the gaze point of the human driver—having been projected out onto the three-dimensional world and then reprojected back to screen coordinates based on the model car’s position and rotation. The purple line shows the future path that the model will take.

The simulated environment was displayed at a 60 Hz frame rate and presented on a display with a horizontal field of view of roughly 70°. Participants were instructed to simply stay on the track—loudspeakers played a “beeping sound” if the participant veered off the (invisible) 3.5-m-width track. The simulator used a raycast vehicle model (as implemented in the Cannon.js JavaScript physics library) to simulate vehicle physics.

We took the centerline of the track as our reference track (see the red line in the right panel of Figure 3) and used this to determine how far along the track both the human and model drivers were positioned. The model car's “track position” in Experiment 2 thus refers to the indexed position on the track centerline that is closest to the vehicle position. Like in Experiment 1, the track positions were used to determine the gaze input for the models from where the human driver was looking at the corresponding track position.

In the analysis of Experiment 2, the first and last curves of each trial were removed (leaving a total of 14 curves). The first curve was removed because the virtual car would at the start accelerate to the automatically kept speed of 10.5 m/s, which was not simulated with the models. The last curve was removed simply to keep the number of left and right curves equal.

### Steering and gaze behavior measurement

Steering control was produced using a Logitech G27 (Logitech, Fremont, CA, USA) steering wheel in Experiment 1 and Logitech G920 (Logitech) steering wheel in Experiment 2. Steering behavior was recorded for each frame of the simulators (60 Hz).

In both experiments, the participants' eye movements were recorded with a binocular head-mounted Pupil Core (Pupil Labs UG haftungsbeschränkt, Berlin, Germany) eye tracker. Eye image data were collected binocularly at 60 Hz, at a resolution of 640 × 480, and a forward-facing camera recorded the scene at 30 Hz, at a resolution of 1920 × 1080 in Experiment 1 and 1280 × 720 in Experiment 2.

**Figure 6.**
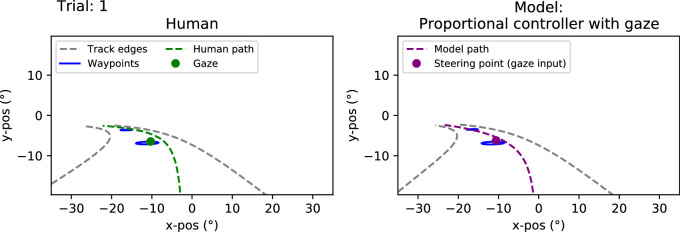
Experiment 2. A sample frame from the Supplementary Movies. The full movies depict the model runs from a first-person perspective side-by-side with the trial from the human driver. *Left panel.* Human driver’s view. The green circle depicts the screen coordinates of the gaze signal as recorded by the eye tracker. The dashed green line depicts the driver’s future path (the actual trajectory they will take). The blue circles depict the visible waypoints and the gray dashed lines the invisible track edges. *Right panel.* The model driver’s view in the corresponding trial and track position. The purple circle depicts the steering point, which in this case is based on the gaze point of the human driver—having been projected out onto the three-dimensional world and then reprojected back to screen coordinates based on the model car’s position and rotation. The purple line shows the future path that the model will take.

The gaze distribution of participants from both experiments is displayed in Figure 4. Gaze behaviour from individual trials is displayed in the Supplementary Movies (available at: https://doi.org/10.6084/m9.figshare.23456540) with sample frames shown in Figures 5 and 6. The Supplementary Movies show the reconstructed first-person view of the human and model drivers with the driver’s gaze and future path overlaid on top—the movies have been reconstructed from the data so they do not display the textures as seen by the human participants (see Figure 2 for sample screenshots of the view that was presented to participants).

### Data modeling methods: Steering controllers

#### Proportional controller

The proportional controller was deployed to test how well steering-by-gaze could be achieved by the simplest means we could imagine, effectively using the simplest linear controller with a one-dimensional input (here: horizontal gaze direction relative to current direction of travel), similar to the much-used 2-point proportional derivative (PD) controllers like those by Salvucci and Gray (2004) and van Maanen, Heiden, Bootsma, and Janssen (2021), but even simpler. Given the horizontal screen coordinate of the steering point, the proportional controller will simply set the target yaw-rate to be proportional to the angular offset of the steering point from the middle of the screen.

To determine the angular offset, the controller is first provided with the steering point (*x*, *z*) in egocentric world coordinates where the z-axis is the straight-ahead direction/locomotor heading of the vehicle and x-axis is the lateral axis, perpendicular to the z-axis and the vehicle’s current position is the origin. Assuming no vehicle roll or pitch, the horizontal screen coordinate *h* is thus:

(1) $$\begin{array}{c} h=arctan(x/z)\; \end{array}$$

The proportional controller sets target yaw-rate *y*′ to

(2) $$\begin{array}{c} y^{'}=k*h\; \end{array}$$

where *k* is the gain parameter.

Note that the proportional controller uses only the horizontal direction of the gaze and does not use any explicit depth information, unlike the pure-pursuit controller.

#### Pure-pursuit controller

The pure-pursuit controller was chosen because it is a simple controller for intercepting waypoints (here: points of fixation on the plane of travel). It has been widely used in vehicle automation as a solution for steering a vehicle through a designated path or a series of designated targets (Samuel et al., 2016).

Pure-pursuit calculates the required constant angular velocity of a moving body/vehicle to intercept a given steering point when traveling at a constant speed (forward velocity in moving observer frame of reference). In other words, given a steering point and some fixed speed, the pure-pursuit controller calculates the target yaw-rate that would allow the vehicle to reach the steering point on a circular trajectory at a constant yaw-rate from the current position. As the target yaw-rate is recomputed at every point in time, the actual resulting trajectory is not necessarily circular.

Mathematical description of the controller is fairly simple. The controller is given a steering point (*x*, *z*) in egocentric world coordinates where again the z-axis is the straight-ahead direction/locomotor heading of the vehicle and x-axis is the lateral axis and the vehicle’s current position is the origin. The target yaw-rate *y*′ that allows the vehicle to reach the steering point on a circular trajectory of constant yaw-rate with speed *v* is

(3) $$\begin{array}{c} y^{'}=k*2*v*x/\left(x^{2}+z^{2}\right)\; \end{array}$$

where *k* is the gain parameter.

Typically, in engineering models, the controller is given a steering point at some constant distance away from the vehicle/robot, but in the present study, we test how the controller performs when given as input gaze landing points in the scene (points of fixation) measured from actual human drivers.

#### Exponential smoothing

For all the models, the target yaw-rate produced by the controllers was exponentially smoothed to produce the actual simulated yaw-rate. The yaw-rate *y*_*t*_ at given time *t* was thus:

(4) $$\begin{array}{c} y_{t}=a*y_{t}^{'}+\left(1-a\right)*y_{t-1}\; \end{array}$$

where *a* is the smoothing factor, $y_{t}^{'}$ is the target yaw-rate given by the controller at time *t*, and *y*_*t* − 1_ is the yaw-rate at the previous time step. The smoothing restricts how fast the virtual vehicle's yaw-rate could change (i.e., when *a* is small, the simulated car cannot instantaneously change direction when the steering point/target yaw-rate changes). Note that in the discrete implementation, the scale of *a* is sensitive to the frame rate (60 Hz in both experiments).

In Experiment 1, the yaw-rate was also clipped to a maximum of 35°/s and a minimum of −35°/s to match the limits set on the human drivers. In Experiment 2, no clipping was performed as no such limits were set on the human drivers.

### Model inputs (steering points)

We refer to the coordinates of a point (in a suitable frame of reference), which can be given to a steering controller as “steering points” (Lappi, 2014). We investigated two kinds of steering points: (a) gaze points from human participants projected as points of fixation on the plane of travel in the (virtual) world and (b) the fixed optical waypoints placed on the plane of travel in the (virtual) world. In the case of the pure-pursuit controller, the steering points were then transformed into egocentric (virtual) world coordinates in reference to the current position and locomotor heading of the driver. In the case of the proportional controller, the steering points were transformed from virtual world coordinates back to (simulated) display coordinates with the only consideration being the angular distance from the current locomotor heading (vertical meridian of the virtual field of view). A more detailed explanation for how the steering points were derived follows below.

#### Points of fixation and gaze direction

For each frame in the data, the gaze direction (calibrated in the head-mounted camera coordinates) provided by the eye tracker was projected from camera coordinates to the display coordinates. The transformation from camera to display coordinates was done by estimating a homography between each frame of the camera and the display via optical screen markers with known screen coordinates (see Figure 2). The display coordinates were then projected to points of fixation in world coordinates of the simulation, that is, interception of the line of sight and the plane of travel, via the known pose (translation and rotation) of the virtual camera. These points of fixation were then associated with the driver's track position at the time of the frame, that is, the closest point on the reference track to the virtual car (in Experiment 1, the reference track is the mean path, and in Experiment 2, it is the circular arc).

For the steering models, the points of fixation on the plane of travel were linearly interpolated, according to the model car's track position. The interpolation was done as a function of track position rather than time in order to avoid accumulation of error over time (i.e., if the model car took a longer path by steering closer to the outer edge than the human, then the gaze points would be given from an increasingly further distance from the model car if the interpolation was done in respect to time). In other words, the models were provided the moment-to-moment gaze direction from where the human driver looked at from the corresponding track position in the trial that was being simulated.

For the proportional controller, the point of fixation was transformed back from three-dimensional world coordinates to simulated display coordinates according to the model car's position and rotation, and the horizontal coordinate was then given to the model as the egocentric gaze direction.

#### Optical waypoints

In addition to running the controllers on the human-derived gaze data, we ran the controllers using the optically available waypoint cues (the blue waypoints in Experiment 1 and white waypoints in Experiment 2) as steering inputs. The location of the closest-to-the-observer waypoint on the track was used as the steering point for the controller until the distance between the optical waypoint and the simulated vehicle became smaller than th*v, where *v* is the speed of the vehicle and *th* a free parameter that represents the time headway (in seconds) when the active steering point is switched to the next optical waypoint on the track.

In the case of the pure-pursuit controller, both the *x*,*z* egocentric world coordinates were provided to the controller. In the case of the proportional controller, only the gaze direction (i.e., the simulated horizontal display coordinate) was given.

### Running the models

In total, we chose four different models to compare, with two different controllers and two different inputs:

(1) Pure-pursuit controlled model with gaze as steering input
(2) Proportional controlled model with gaze as steering input
(3) Pure-pursuit controlled model with optical waypoints as steering input
(4) Proportional controlled model with optical waypoints as steering input

The models were then run for each frame of each participant and trial in both experiments. The starting position and rotation of the model car were set according to the actual participant's position and rotation, but beyond that, the models had to steer through the entire track in both experiments without any manual corrections. Vehicle physics were not simulated for either experiment.

Given that they were always given the same steering inputs, the between-trial variation in optically guided model performance was only determined by their starting position and direction. The gaze-guided models, however, were given the actual participant's gaze point as their input at each point in time for each trial (however, correspondence of the point in time was determined by the distance traveled on the track, rather than the index of the frame).

If more than 10% of the gaze points in a trial were more than −2 degrees above the horizon, the trial was excluded from the data. This was done as gaze points near the horizon would be projected very far into the distance, or even behind the driver (for points above the horizon), making point of fixation estimation unreliable—as previously mentioned, the rationale for the projection was to ensure the gaze input would refer to the same location in the (virtual) world regardless of the model vehicle’s pose and position. In Experiment 2, one participant was excluded from the data entirely because of this (leaving a final *N* = 15).

### Parameter optimization

Before running the final models, we optimized the values of the parameters (gain *k* and smoothing factor *a* in case of the gaze-guided models and *k*, *a*, and time headway *th* in case of the waypoint-guided models) for the four models to minimize the mean distance error from the human trajectories. Although the values of the parameters were kept the same for all participants, the models were run separately for each human participant with the minimized distance error being determined as the grand mean of the participant-wise means (i.e., the mean distance between the human and model positions in respect to closest track position, pooled across all participant trials).

The optimization was done by first performing a grid search through a specified search space (*th* range: 0.1–0.8, *k* range: 0.9–3, *a*: 0.05–1) to find a rough initial guess of the optimal values. The minimums found by the grid search were then used as the initial guesses for the Nelder–Mead algorithm that was used to find the approximate (local) minimums. See Table 1 for the resulting values.

**Table 1.** Parameter values resulting from the optimization for the four models: resulting from two different inputs and control laws.

Model		Parameters		
Input	Controller	Gain *k*	Smoothing factor *a*	Waypoint switch *th*
Gaze	Pure-pursuit	1.24	0.06	N/A
Gaze	Proportional	2.23	0.06	N/A
Waypoints	Pure-pursuit	1.25	0.06	0.59
Waypoints	Proportional	2.83	0.16	0.25

To avoid overfitting, and to test how well the parameter values would generalize, the optimization was done only for Experiment 1 with the resulting values used also in Experiment 2. As the participants in the two experiments were not the same and to avoid overfitting for individual participants, the optimization was performed with all data pooled together.

## Results

To evaluate the performance of each model, we calculated three different metrics: (a) the distance between the model-produced trajectories and the human-produced trajectories at each track position (determining a participant-wise mean error and grand mean error for the model) to measure the positional difference in the real and simulated trajectories, (b) the total time off track (beyond the road boundaries) as percentage of trial duration to measure, and (c) the Pearson correlation coefficient between the human- and model-produced yaw-rates (yaw-rate correlation) to measure the similarity in steering. All the metrics were measured within participants and then averaged across participants.

In addition, we estimated trajectory correlations at three different points to better estimate if and how well the models could explain within-participant variation in the trajectories. Because of the sequential time-course nature of the steering trajectories, it is possible that the correlation decreases over time as the distance from the starting conditions increases, or that the models capture steering at different times with different degrees of success. To test this in both experiments, we took three points on the track (representing the beginning, middle, end) and calculated the correlation between the human- and model-produced trajectory coordinates for each participant over all trials. In Experiment 1, the three points used were the waypoints (labeled “Trajectory correlation” WP 1, WP 2, WP 3), whereas in Experiment 2, the exact beginning, middle, and the end of the track (first and last curves omitted) were used (labeled “Trajectory correlation” Beginning, Middle, End). This analysis provided an estimate of whether the variance in the model-produced trajectories correlated with the variance in the actual trajectories.

### Experiment 1: Modeling results

**Figure 7.**
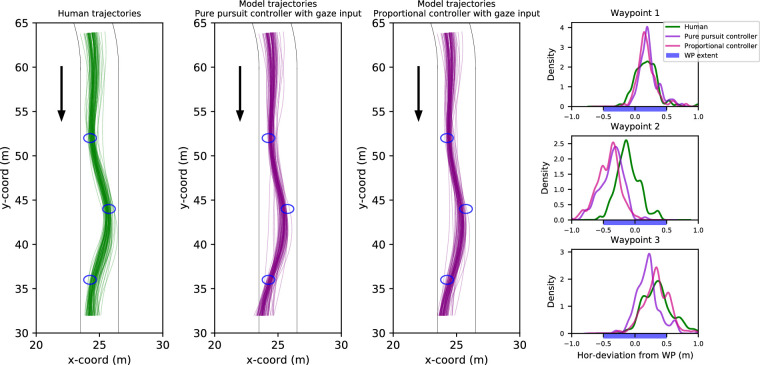
*First three columns*. Human and (gaze-input) model trajectories in Experiment 1. Black lines indicate visible edgelines of the track and blue circles the visible waypoints. Human drivers’ trajectories are depicted in green and model trajectories in purple. *Rightmost column*. The kernel density estimates depict the distribution of deviations from each of the three waypoints for the human and model drivers when passing over the waypoint in question.

The trajectories produced by the gaze-guided models are displayed in Figure 7. It can be seen that these models are generally successful in steering via each of the waypoints. The density plots (Figure 7, right panels) show that the models cross Waypoints 1 and 3 at a similar location to the Human trajectories. Waypoint 2, however, is only clipped by the models, with the density shifted nearer to the road center when compared to Human trajectories. This “understeering” at Waypoint 2 may potentially be the result of look-ahead fixations to Waypoint 3, attracting the models to steer early in the direction of Waypoint 3.

**Figure A1.**
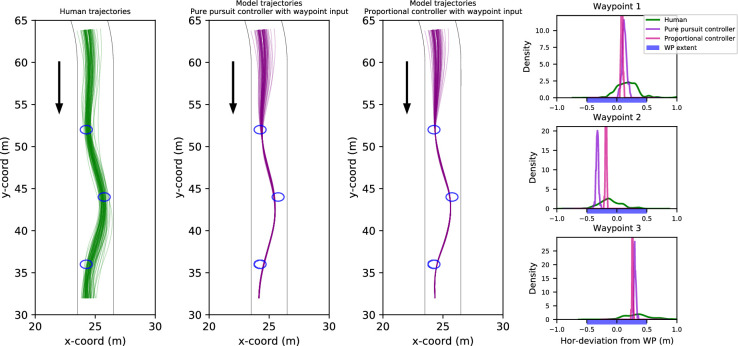
*First three columns*. Human and (waypoint-input) model trajectories in Experiment 1. Black lines indicate visible edgelines of the track and blue circles the visible waypoints. Direction of travel is from top to bottom. Human drivers’ trajectories are depicted in green and model trajectories in purple. *Last column*. Density estimations of horizontal deviations from each of the three waypoints for the human and model drivers when passing over the waypoint in question.

Trajectories produced by the waypoint-guided models are displayed in Figure A1. For both controllers, the trajectories universally pass through the waypoints. As the trials only vary in their starting position and direction, the trajectories quickly converge with very little variation between them.

**Figure 8.**
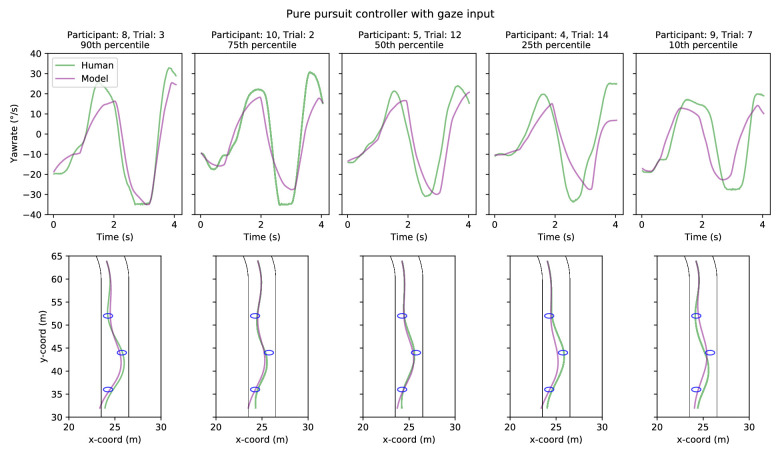
Five sample trials comparing model (pure-pursuit controller with gaze input) and human performance in Experiment 1. Sample trials have been chosen on the basis of yaw-rate correlation between the model and human drivers: choosing the 90th, 75th, 50th, 25th, and 10th percentile trials. *Top row*. Model (purple line) and human (green line) yaw-rates as a function of time. *Bottom row*. Model (purple line) and human (green line) trajectories.

**Figure 9.**
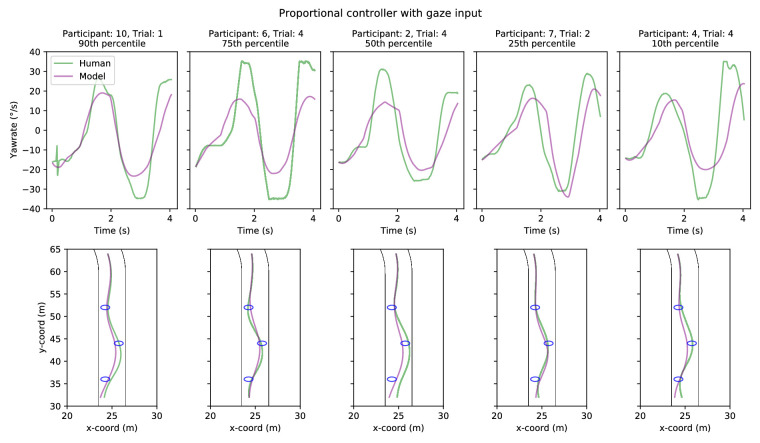
Five sample trials comparing model (proportional controller with gaze input) and human performance in Experiment 1. Sample trials have been chosen on the basis of yaw-rate correlation between the model and human drivers: choosing the 90th, 75th, 50th, 25th, and 10th percentile trials. *Top row*. Model (purple line) and human (green line) yaw-rates as a function of time. *Bottom row*. Model (purple line) and human (green line) trajectories.

**Figure 10.**
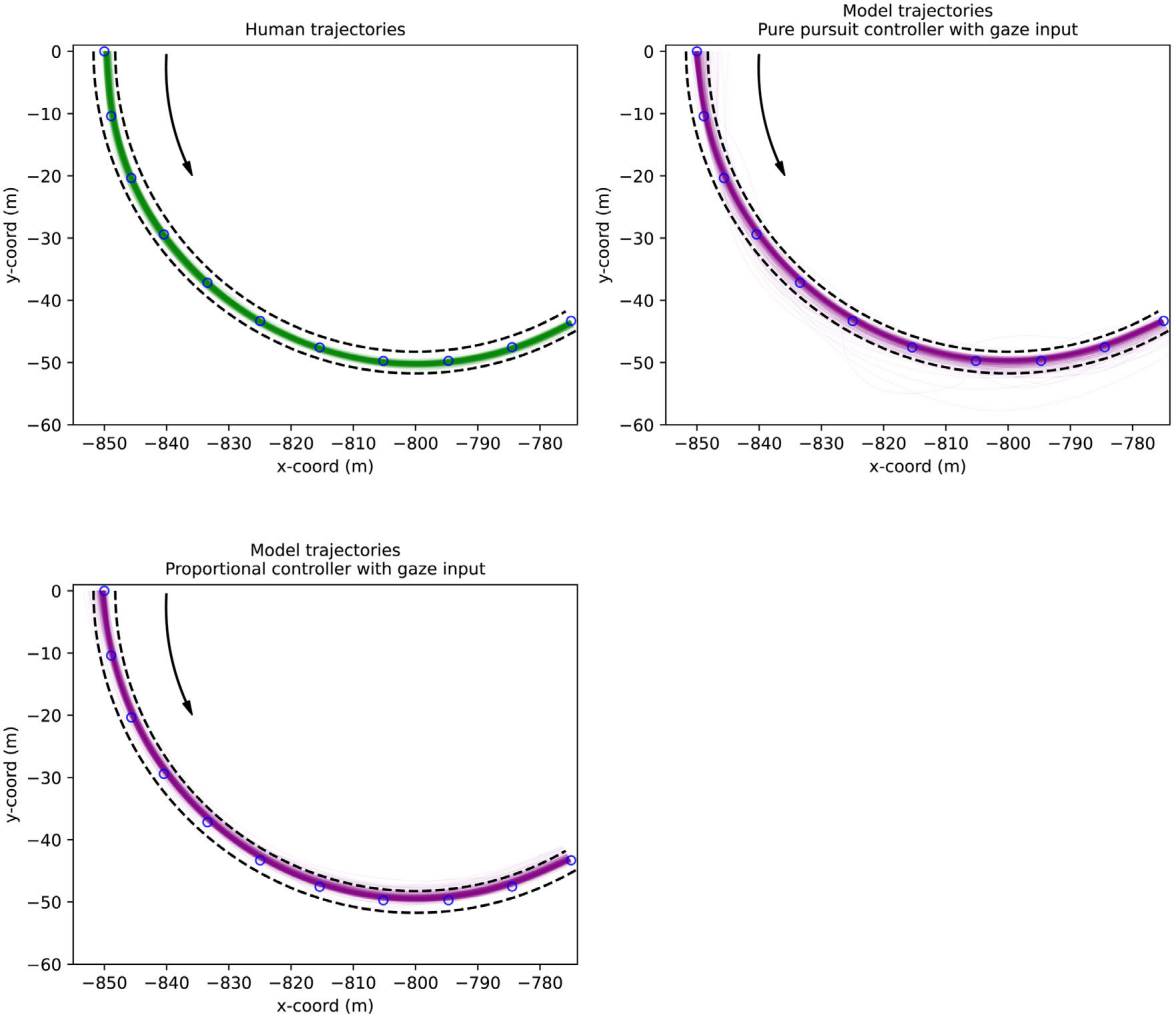
Human and (gaze-input) model trajectories in Experiment 2. All left- and right-turning curves (14 total) have been transformed and projected to a single curve. Dashed black lines indicate invisible edge lines of the track and blue circles the visible waypoints. *Upper left panel.* Human trajectories in green. *Upper right panel.* Model trajectories produced by the pure-pursuit controller with gaze input depicted with purple lines. The model ends up spending roughly 1.5% of the time off track, with most “offtrack time” likely being caused by gaze being off the track—due to the inclusion of depth information, the pure-pursuit controller is also sensitive to changes in the vertical position of gaze, unlike the proportional controller (gazing over the horizon is especially problematic with the projected point of fixation being projected behind the driver instead). *Lower left panel.* Model trajectories produced by the proportional controller with gaze input depicted with purple lines. The model tends to steer more toward the inner edge—likely caused by the controller parameters being derived from Experiment 1, which has resulted in high gain.

**Figure 11.**
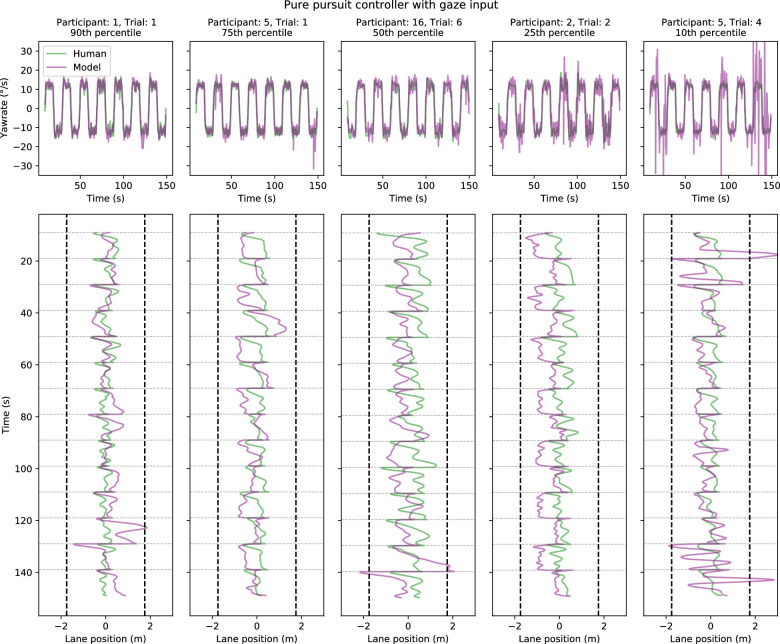
Five sample trials comparing model (pure-pursuit controller with gaze input) and human performance in Experiment 2. Sample trials have been chosen on the basis of yaw-rate correlation between the model and human drivers: choosing the 90th, 75th, 50th, 25th, and 10th percentile trials. The time series depict performance across 14 bends in each single trial (with the first and last curves omitted from the total of 16 bends as described in Methods). *Top panels.* Model (purple lines) and human (green lines) yaw-rates as a function of time. *Bottom panels.* The corresponding lane positions (on the x-axis) as a function of time (y-axis). The lane position indicates the distance from the centerline, with positive values indicating the driver is more toward the outer edge rather than the inner edge. The vertical dashed lines indicate the track edges. The faint gray horizontal lines indicate the locations where the sign of the road changes (i.e., when the driver passes from a left-turning curve to a right-turning curve or vice versa)—this also explains the sudden changes in lane position as what was the outer edge of the previous curve becomes the inner edge of the new curve and vice versa.

**Figure 12.**
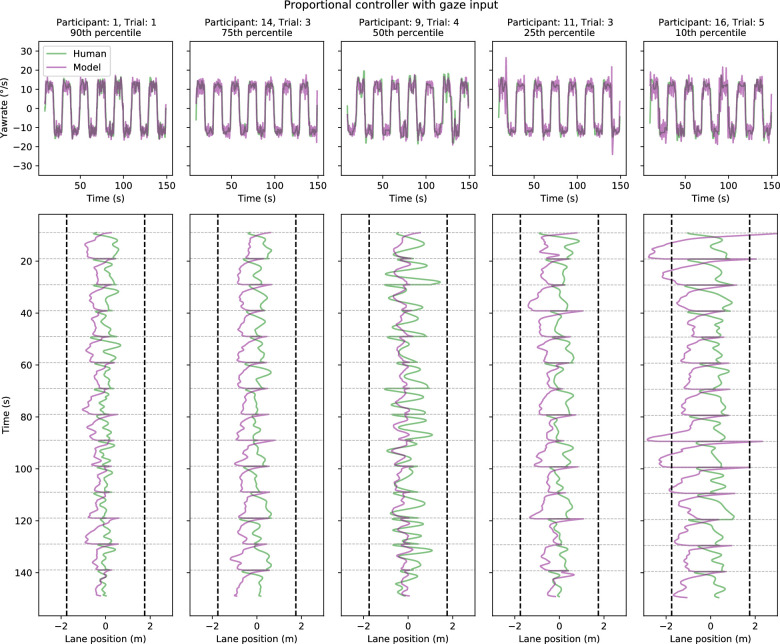
Five sample trials comparing model (proportional controller with gaze input) and human performance in Experiment 2. Sample trials have been chosen on the basis of yaw-rate correlation between the model and human drivers: choosing the 90th, 75th, 50th, 25th, and 10th percentile trials. The time series depict performance across 14 bends in each single trial (with the first and last curves omitted from the total of 16 bends as described in Methods). *Top panels.* Model (purple lines) and human (green lines) yaw-rates as a function of time. *Bottom panels.* The corresponding lane positions (on the x-axis) as a function of time (y-axis). The lane position indicates the distance from the centerline, with positive values indicating the driver is more toward the outer edge rather than the inner edge. The vertical dashed lines indicate the track edges. The faint gray horizontal lines indicate the locations where the sign of the road changes (i.e., when the driver passes from a left-turning curve to a right-turning curve or vice versa)—this also explains the sudden changes in lane position as what was the outer edge of the previous curve becomes the inner edge of the new curve and vice versa.

**Figure A2.**
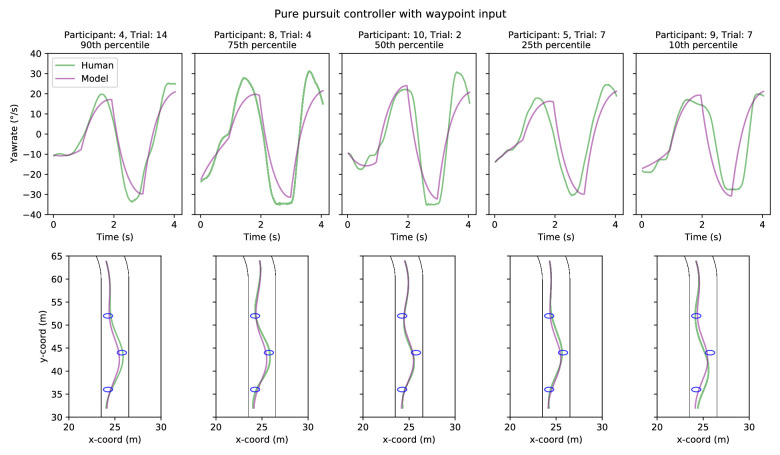
Five sample trials comparing model (pure-pursuit controller with waypoint input) and human performance in Experiment 1. Sample trials have been chosen on the basis of yaw-rate correlation between the model and human drivers: choosing the 90th, 75th, 50th, 25th, and 10th percentile trials. *Top row*. Model (purple line) and human (green line) yaw-rates as a function of time. *Bottom row*. Model (purple line) and human (green line) trajectories.

**Figure A3.**
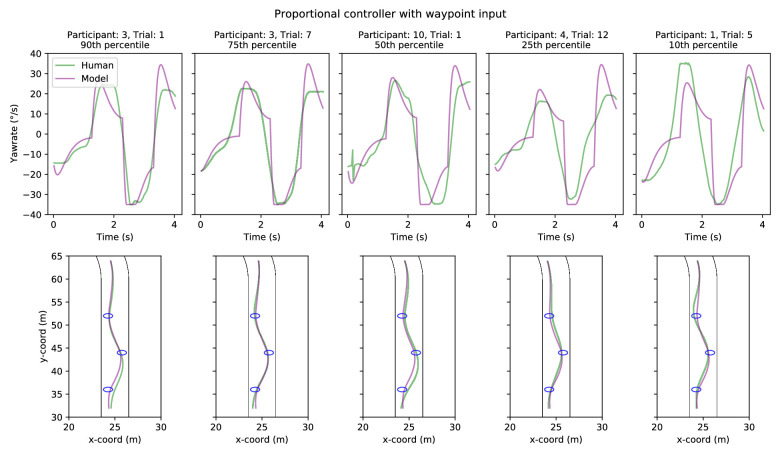
Five sample trials comparing model (proportional controller with waypoint input) and human performance in Experiment 1. Sample trials have been chosen on the basis of yaw-rate correlation between the model and human drivers: choosing the 90th, 75th, 50th, 25th, and 10th percentile trials. *Top row*. Model (purple line) and human (green line) yaw-rates as a function of time. *Bottom row*. Model (purple line) and human (green line) trajectories.

Example individual yaw-rates and trajectories for the gaze-guided models are shown in Figures 8 and 9. The yaw-rate time series for both controllers (Figures 8 and 9, upper panels) appears quite similar between the human and model drivers, with comparable amplitudes and timing for changes in steering/yaw-rate across all examples. The displayed example trials were selected based on the goodness of fit (and lack thereof). The trajectories (Figures 8 and 9, lower panels), on the other hand, demonstrate more variation in fit, though the models still cross or at least clip all of the waypoints (interestingly, the 10th percentile example in Figure 8 shows the human driver failing to cross two of the waypoints). Sample trajectories for the optical waypoint-guided models are displayed in Figures A2 and A3, but there is effectively no difference between individual trials as far as model behavior goes.

A summary of the different performance metrics can be seen in Table 2, and participant-wise results are reported in Tables A1–A4. The grand mean error for both the gaze-guided pure-pursuit and proportional controllers was around 0.2 m and mean yaw-rate correlation coefficients were around 0.85 (for both controllers). No significant differences were found between the performance of the two gaze-guided models (paired samples *t*-test for participant mean errors, *t* = 1.51, *p* = 0.16; paired samples *t*-test for *z*-transformed yaw-rate correlations, *t* = 1.0, *p* = 0.34). The time spent off track was minimal for both models; we found no statistically reliable difference between the two models (Binomial test, *p* = 1.0). Overall, both of the models steered similarly to the human drivers, although some individual modeled trials fail to pass over the waypoints (though that is also true for some of the human trials—not necessarily in the same trials, however).

**Table 2.** Summary table of model performance in Experiment 1. The mean yaw-rate correlation for all the models is high (∼0.85 for the gaze-guided models), indicating similar patterns of steering. The grand mean error is approximately 0.2 m for all the models with minimal time spent off track. The grand mean error has been calculated as the mean of participant-wise mean errors across trials, and the mean offtrack proportion was similarly calculated by determining the proportion spent off the invisible edgelines over all trials within each participant and taking the mean across participants. The trajectory correlations have been calculated by taking the horizontal position of the human and model driver as they pass each waypoint and calculating the Pearson’s correlation coefficient for each participant—the reported value is the retransformed mean of the participant-wise Fisher *z*-transformed correlations. Note that the models start from the same position as where the human driver was located, which can lead to spurious correlation at the early WPs (in the waypoint-guided models, the trials only differ by their starting conditions and then converge to essentially a single trajectory, likely explaining the decrease in trajectory correlation).

Model		Performance			Trajectory correlation		
Input	Controller	Mean yaw-rate *r*	Grand mean error (m)	Mean offtrack time (%)	WP 1	WP 2	WP 3
Gaze	Pure-pursuit	0.85	0.22	0.32	0.24	0.20	0.48
Gaze	Proportional	0.84	0.21	0	0.15	0.24	0.47
Waypoints	Pure-pursuit	0.92	0.17	0	0.53	0.31	0.04
Waypoints	Proportional	0.88	0.16	0	0.36	0.26	0.04

**Table 3.** Summary table of model performance in Experiment 2. The mean yaw-rate correlation is above 0.9 for all the models, indicating similar patterns of steering. The grand mean error ranges from approximately 0.3 to 0.7 m (for context, the path width was 3.5 m) with a small amount ( 1.5%) of time spent off track for the gaze-guided models and none for the optical waypoint-guided models. The grand mean error has been calculated as the mean of participant-wise mean errors across trials, and the mean offtrack proportion was similarly calculated by determining the proportion spent off the invisible edgelines over all trials for each participant and taking the participant-wise mean. The trajectory correlations have been calculated by taking the lane position of the human and model driver as they pass each position and calculating the Pearson’s correlation coefficient for each participant—the reported value is the retransformed mean of the participant-wise Fisher *z*-transformed correlations.

Model		Performance			Trajectory correlation		
Input	Controller	Mean yaw-rate *r*	Grand mean error (m)	Mean offtrack time (%)	Beginning	Middle	End
Gaze	Pure-pursuit	0.95	0.51	1.49	0.19	0.06	−0.07
Gaze	Proportional	0.96	0.71	1.68	0.03	0.10	−0.15
Waypoints	Pure-pursuit	0.98	0.37	0	−0.03	0.11	−0.10
Waypoints	Proportional	0.91	0.27	0	−0.03	−0.32	−0.04

We estimated correlations between the model-produced and individual human trajectories by looking at the driver’s signed horizontal position when passing each of the waypoints (i.e., at the track position where the reference trajectory was closest to the waypoint in question). At least as far as WPs 1 and 2 are concerned, we found no strong correlation between the model trajectories and the individual human trajectories. Curiously, there was a moderate correlation (retransformed mean of Fisher *z*-transformed Pearson’s correlation coefficient was 0.48 for the pure-pursuit controller and 0.47 for the proportional controller) at WP 3 with both controllers, resulting in 10 of 11 participants having a positive correlation (Binomial test, *p* = 0.006). We have no obvious explanation for this pattern, but it could have been due to some behavioral change (e.g., the result of fewer look-ahead fixations past the second waypoint and gaze being directed more consistently in the direction of travel near WP 3).

### Experiment 2: Modeling results

**Figure A4.**
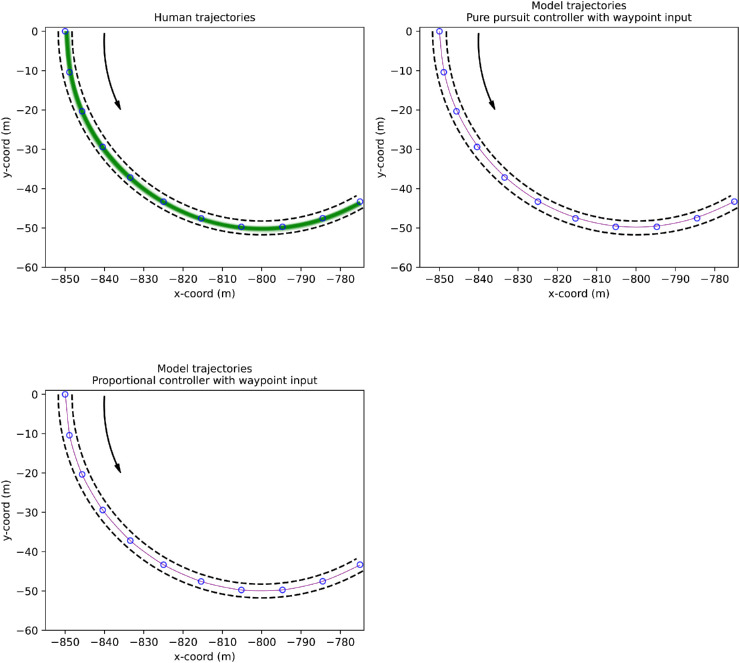
Human and (waypoint-input) model trajectories in Experiment 2. All left- and right-turning curves have been transformed and projected to a single curve. Dashed black lines indicate invisible edgelines of the track and blue circles the visible waypoints. *Upper left panel.* Human trajectories in green. *Upper right panel.* Model trajectories produced by the pure-pursuit controlled with gaze input depicted with purple lines. *Lower left panel.* Model trajectories produced by the proportional controlled with gaze input depicted with purple lines.

The trajectories produced by the gaze-guided models (using parameters based on Experiment 1) are displayed in Figure 10. Both controllers appear to steer successfully within the track most of the time (∼98%) with some exceptions: The proportional controller sometimes exhibits oversteering and leaves the track via the inner edge, whereas the pure-pursuit controller sometimes leaves via the inner and outer track edges. Trajectories produced by the waypoint-guided models are displayed in Figure A4. The waypoint-guided models steer successfully through the waypoints without leaving the track (and with little variation between trials), and there are no clear differences in performance between the two controllers (Table 3).

**Figure A5.**
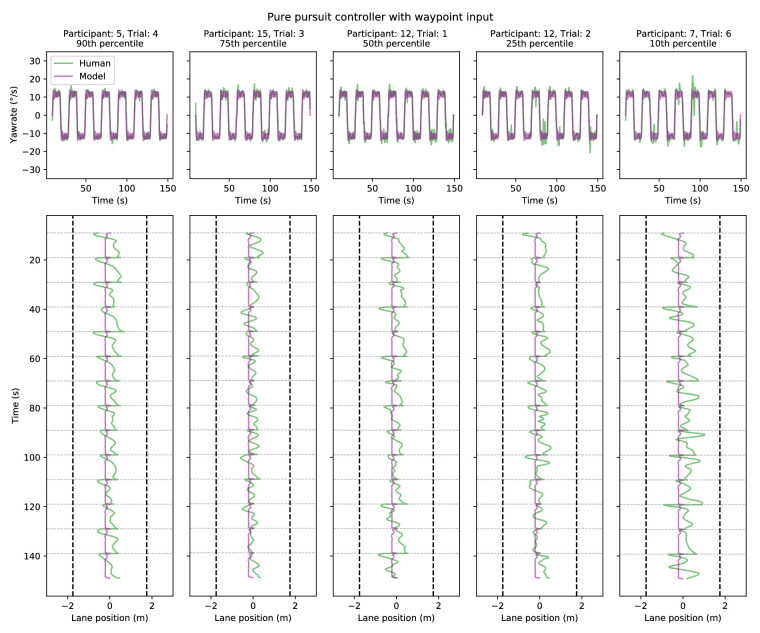
Five sample trials comparing model (pure-pursuit controller with waypoint input) and human performance in Experiment 2. Sample trials have been chosen on the basis of yaw-rate correlation between the model and human drivers: choosing the 90th, 75th, 50th, 25th, and 10th percentile trials. *Top panels.* Model (purple line) and human (purple line) yaw-rates as a function of time. *Bottom panels.* The corresponding lane positions (on the x-axis) as a function of time (y-axis). The lane position indicates the distance from the centerline with positive values indicating the driver is more toward the outer edge rather than the inner edge. The vertical dashed lines indicate the track edges. The faint gray horizontal lines indicate the locations where the sign of the road changes (i.e., when the driver passes from a left-turning curve to a right-turning curve or vice versa).

**Figure A6.**
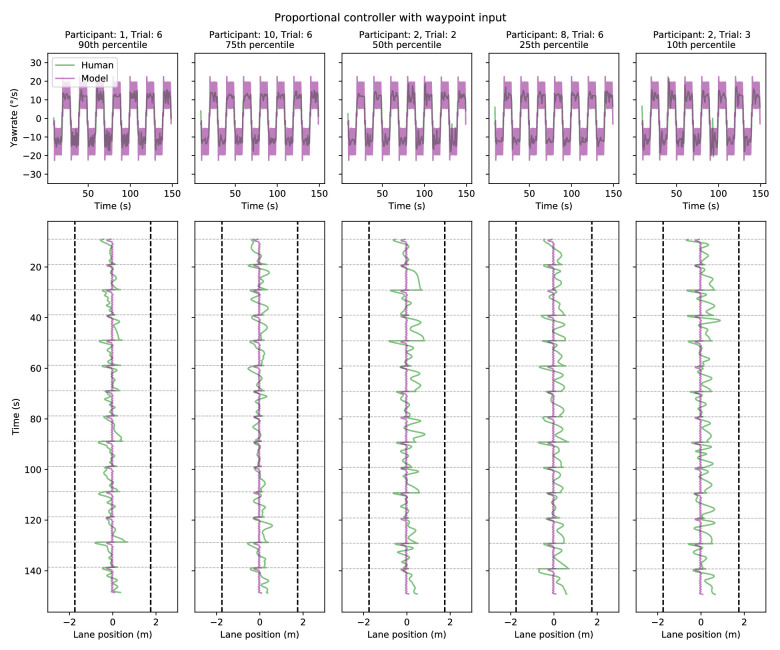
Five sample trials comparing model (proportional controller with waypoint input) and human performance in Experiment 2. Sample trials have been chosen on the basis of yaw-rate correlation between the model and human drivers: choosing the 90th, 75th, 50th, 25th, and 10th percentile trials. *Top panels.* Model (purple line) and human (purple line) yaw-rates as a function of time. *Bottom panels.* The corresponding lane positions (on the x-axis) as a function of time (y-axis). The lane position indicates the distance from the centerline with positive values indicating the driver is more toward the outer edge rather than the inner edge. The vertical dashed lines indicate the track edges. The faint gray horizontal lines indicate the locations where the sign of the road changes (i.e., when the driver passes from a left-turning curve to a right-turning curve or vice versa).

Sample yaw-rates and trajectories are shown in Figures 11 and 12. Similar to Experiment 1, the yaw-rate time series of the model and human drivers look very similar, but with occasional spikes in the model-produced yaw-rates (corresponding with the spikes in the gaze signal). Despite the similarity in yaw-rates, the steering input still leads to differences in the resulting trajectories. Sample trajectories for the waypoint-guided models are displayed in Figures A5 and A6, but there are effectively no differences between individual trials as far as waypoint-guided model behavior goes.

The summary of the different performance metrics can be seen in Table 3 and participant-wise results can be seen in Tables A6–A9. The mean error for the gaze-guided pure-pursuit controller was 0.51 m and 0.71 m for the proportional controller. The difference in performance in terms of mean error is significant (paired samples *t*-test, *t* = −3.36, *p* = 0.005) with the difference likely being explained by the proportional controller steering closer to the inner edge of the track. Yaw-rate correlations were even higher than in Experiment 1, but we found no significant difference between the two gaze-guided models (0.95 and 0.96 mean correlations for the gaze-guided controllers; paired samples *t*-test, *t* = −1.20, *p* = 0.25). In the majority of trials, the models were able to successfully steer through the entire track, but they did occasionally veer off (1.49% of the total runtime in the case of the pure-pursuit controller and 1.68% in the case of the proportional controller); we found no significant difference between the two models (binomial test, *p* = 1.0). The pure-pursuit controller failed one trial entirely by veering off more than 10 m away from the edge lines.

We estimated the correlation between the model and individual human trajectories by comparing the model and human driver’s signed lane positions when passing the very beginning of the track (i.e., the beginning of the second bend after accounting for the omission of the first bend from the analysis), the middle of the track, and the very end of the track. There was no correlation between the human trajectories and the model trajectories at the designated positions (Beginning, Middle, End) with any of the models. There was no replication of the moderate correlation found later in trials (WP 3) as in Experiment 1. It appears, then, that the models fail to capture the variability of the individual trajectories of the human drivers.

## Discussion

The aim of this study was to investigate the relationship between steering and gaze behavior by examining how different computational steering models behave when only gaze direction is given as the models' steering input. Each controller was made to steer-by-gaze without access to any visual information from the environment (“steering based on *where* you are looking,” not “steering based on *what* you see”) in order to gauge whether successful steering can be produced from gaze alone.

While the experimental designs were highly controlled, there was still considerable room for variability in the timing and placement of gaze. For example, in the slalom task of Experiment 1, the participants could look at any of the three waypoints or onto the ground between them, or simply fixate at the horizon, at any particular instant. With around three fixations a second, gaze could have shifted between any of these zones in a wide variety of possible patterns, many of which would not have supported successful steering (e.g., looking further ahead than the most immediate waypoint or switching between waypoints too soon).

The observed gaze behavior, however, was sufficiently compatible with the assumptions of the the gaze-guided models to allow them to successfully (for the most part) steer through the different tracks employed in Experiment 1 and Experiment 2 (using parameters optimized for Experiment 1). The average trajectories of the models appeared to be similar to human drivers, with the difference in position being 0.4 m on average (the path widths were 3 and 3.5 m for the two experiments). While the position of the driver in the world is important from the perspective of the steering task (i.e., ensuring the driver physically crosses the intended goal), the actual execution of steering is via changes in the direction of travel (the yaw-rate). Overall, the models were able to produce successful steering across two different scenarios using gaze information alone, producing steering patterns that were similar to those of human drivers. This suggests that the human driver's gaze placement is at least *in principle* able to facilitate steering (i.e., gaze contains sufficient information to guide steering).

In terms of general performance (yaw-rate correlation, grand mean error, and mean offtrack time), there did not appear to be major differences between the two controllers. In Experiment 2, the difference in mean error was higher for the gaze-guided proportional controller than for the gaze-guided pure-pursuit, but detailed examination showed that the latter had more “extreme” failures (causing some trajectories to travel far from the road), whereas the former more often drifted off the inner edge, cutting the corners (which could have been reduced using a smaller gain parameter). The difference was likely the result of the pure-pursuit controller being sensitive to depth information and thus having additional sources of noise. When the inputs were waypoints (rather than gaze), both controllers produce extremely similar trajectories with no variation between trials—which is to be expected given that the models are given the same steering points with just slightly different initial conditions.

### Limitations and future directions

We found that the pure-pursuit and proportional controller models performed similarly, despite relying upon different inputs (only the pure-pursuit controller made use of depth/eye height information). Distinguishing between the performance of these types of steering models may require going beyond the relatively simple steering tasks used in the present experiments. Future work will need to test the models further to see whether they can still steer-by-gaze in more challenging situations such as when speeds vary, tracks have obstacles to avoid, or there are intersections/multiple paths to choose from. Steering models with additional parameters and/or sensory information may well be required to capture human behavior in these circumstances.

We observe that while the gaze-guided models produce significant variation in the trajectories, that variation does not correlate well with the variation in the trajectories of the human drivers. In other words, while the variance in gaze produces variance in the trajectories, the present models do not capture the variance in human behavior. There are several potential (not mutually exclusive) explanations for this mismatch, which we cannot presently tease apart but could guide future modeling efforts:

i Measurement noise (e.g., calibration bias) would produce variation in the steering points fed to the models that obviously would not affect human steering behavior.
ii The models treat every point of fixation as a potential steering point, so when humans occasionally make glances that are decoupled from steering (e.g., look-ahead fixations; Lehtonen, Lappi, Koirikivi, & Summala, 2014; Schnebelen, Lappi, Mole, Pekkanen, & Mars, 2019; Mole et al., 2021), the model nevertheless steers toward these points.
iii The models are optimized to a single set of parameters that are used for every participant’s steering, so if there is systematic individual variation in the way steering is coupled with (and/or occasionally decoupled from) gaze, this will not be captured.
iv The models use a single steering point, which is the point of gaze, as the sole information to steer by, which means the vast majority of visual information in the visual field (i.e., all of peripheral vision) is unused, despite the plausible use of that information by humans (note, however, that most psychological steering models in the literature tend to rely on a single steering point; Lappi, 2014).
v The models have no track memory, or other “cognitive” top-down inputs, while humans can learn and remember track characteristics with repetition (Tuhkanen et al., 2019; Tuhkanen et al., 2021).

Capturing such additional sources of variation (individual differences in gaze strategy, full retinal input, memory of the track layout) would be desirable for more sophisticated modeling of steering control mechanisms in humans.

While the performance of the models was largely successful, it should be noted that they did occasionally fail. On detailed inspection, many of these failures appear to be related to periods when the gaze input falls near the horizon, which (due to foreshortening in the optical projection) results in the steering points being projected extremely far into the distance (or even behind the driver if the gaze is above the horizon). Despite every effort being taken to ensure well-calibrated gaze data, some of these observations could be the result of bias in the calibration of the eye tracker. It is also possible that occasionally, the participants genuinely looked into the distance and not where they were going. The models had no allowance for the occasional decoupling of steering from gaze, while in reality, humans do seem to be able to do this.

Some assumptions have been made in the modeling approaches presented here that could be challenged. The present investigation was based on the following theoretical premises: If the oculomotor system maintains information about the current direction of gaze relative to the direction of locomotor heading, and possibly depth information about the current distance to the point of fixation (by whatever means: perceptual, cognitive, motor), and if this information was passed to steering control, then this information could be useful as “efference copy” to perform steering by gaze. Even determining the visual angle of gaze relative to heading (horizontally, parallel to the plane of travel) may not be trivial, especially during natural locomotion (e.g., walking or running) where the eye, head, body, and vehicle may all point in different directions (and there may be no convenient visual reference like the horizon). Whether the model assumptions hold—that is, whether the oculomotor system in fact retains such gaze-vector-to-heading, or distance-to-point-of-fixation (or equivalently gaze-to-horizontal angle plus eye height) information, and whether such information is passed to and used by locomotor control systems—remains beyond the scope of this article and should be independently investigated. Also, we do not of course claim that even if these assumptions were justified, other information would not also be passed to steering control (i.e., that humans would steer-by-gaze only).

The two steering-by-gaze control strategies that we investigated were the simplest effective ones (with fewest parameters) that we found: using egocentric gaze direction for proportional control and using egocentric direction and distance of the point of fixation for pure-pursuit control. The rationale was that the simpler the controller, the more the simulations would be a test of the underlying control signal. It is worth emphasizing that there is no suggestion that these models reflect the actual processing performed by the human brain (and would most likely fail in more complex task settings), but we do hope they are at least somewhat cognitively plausible from the perspective that their control inputs are derived directly from where the human drivers look. In the future, more sophisticated steering control strategies (e.g., model predictive control) could be implemented with an aim to increase fidelity as well as generalizability.

We tested two different controllers to ensure some degree of robustness to the investigation and to determine whether steering by gaze control would be sensitive to the specifics of the control law or its parameterization. We found that both models were able to steer through the S-bends (Experiment 2) using parameters estimated from the slalom task (Experiment 1). Note, however, that we used fairly straightforward steering tasks—and generalization is from the arguably more challenging slalom task to the easier (though longer) S-bend task. More complex environments would likely challenge these simple models and therefore could necessitate more sophisticated control models that take into consideration the fusion of signals from multiple information sources. And even if the models are able steer via gaze, it of course does not mean that human drivers necessarily do so, only that the information or some of the information required for steering in principle is there.

Future models should look into multisensory integration in both gaze control and steering. Note that the possible use of additional sources of information should not necessarily be considered mutually exclusive, and each might play a role to different extents depending on the strength, quality, and variability of other sources, making the “true” steering mechanisms difficult to disentangle when examining steering and gaze behavior. It might be valuable to also account for possible differences in timing between gaze and steering in a more sophisticated manner beyond simple smoothing.

In addition to using the measured gaze data as inputs, we did also use optically specified waypoint markers along the track (which were visible to the participants and visually designated the path they were meant to follow) as control inputs. This was in order to see how well the models performed in principle when given the “best possible” path information (which is the usual type of input they are designed for, i.e., the optical input, steering-by-what-is-there compared to steering-by-gaze).

### Conclusions: Why do we look where we look?

Previous experiments have shown that gaze reliably anticipates steering by “picking up” steering points in the direction of locomotion (waypoints with approximately a 1-s lead time and a 2-s time headway), with steering actions coupled to gaze control (Wilkie, Kountouriotis, Merat, & Wann, 2010; Lappi et al., 2020; Tuhkanen et al., 2021; Mole et al., 2021). But do humans really need to bring steering points into “foveal vision” to use them for visual guidance? Most often, this gaze control pattern tends to be discussed in terms of the (debatable) function of directing foveal vision to successive targets in the environment. That is, the rationale for different gaze control strategies is implicitly given in terms of being able to resolve visual detail at the point of gaze: We look at task-relevant visual targets in order to see them clearly. In this vein, the natural assumption would be that the purpose of the visual strategy of looking where you are going is to see in sharp detail where you are going (i.e., to observe the path that one desires to traverse). This might not be as straightforward of an explanation as it sounds, however.

By looking at an object or location, we bring it to central vision. Usually, the reason we need to bring objects into central vision is to resolve fine detail (small texture elements that cannot be resolved in the periphery due to crowding; Wolfe et al., 2017; Vater, Wolfe, & Rosenholtz, 2022). But during locomotion, when we are moving our whole body to intercept an object, performance can be supported by blurred or peripheral vision. For example, high-speed sports can be performed under conditions of experimental blurring (Mann, Abernethy, & Farrow, 2010) and driving is a task that can be carried out using peripheral vision (in case of foveal degeneration; Lamble, Summala, & Hyvärinen, 2002)—and even when visual information around the point of gaze is withheld, the coordination pattern of looking where you steer persists (Tuhkanen et al., 2019; Tuhkanen et al., 2021). So, experiments have shown that high-resolution central vision is not in fact critical for successful steering and interception. Why do humans do it then? Why do humans generally “look where they are going” in visually controlled locomotion?

The control of gaze, when directed to a viable steering point, in itself, contains information related to the direction and distance of that steering point. This information could be used to steer-by-gaze where the oculomotor system “designates” via gaze deployment a visual direction or a point in the world, as a steering point for the locomotor system. Designating a fixed waypoint in the world to be intercepted was labeled “the waypoint identification hypothesis” in Lappi and Mole (2018).

This raises the interesting possibility that looking where you are going is not so much about seeing where you are going (i.e., fulfilling the need for bottom-up visual input), but part of an embodied “do it where I'm looking” (Ballard, Hayhoe, Li, & Whitehead, 1992) coordination pattern where the eyes, as opposed to just vision, lead the rest of the body.

## Supplementary Material

Supplement 1

## Appendix

**Table A1.** Participant-wise model results table from Experiment 1 with a pure-pursuit controller and gaze inputs.

	Performance			Trajectory correlation			
Participant	Yaw-rate correlation	Mean error (m)	Offtrack time (%)	WP 1	WP 2	WP 3	Excluded trials
1	0.75	0.27 (*SD* = 0.19)	0	0.62	0.25	−0.45	0
2	0.88	0.33 (*SD* = 0.33)	0	0.68	−0.02	0.78	0
3	0.88	0.19 (*SD* = 0.18)	0	−0.19	0.53	0.81	0
4	0.80	0.25 (*SD* = 0.24)	0.58	0.34	0.22	0.20	1
5	0.91	0.12 (*SD* = 0.11)	0	0.60	0.19	0.59	0
6	0.83	0.16 (*SD* = 0.18)	0.28	0.31	0.26	0.54	0
7	0.88	0.16 (*SD* = 0.15)	0.25	0.05	−0.10	0.44	0
8	0.90	0.2 (*SD* = 0.14)	0.49	−0.05	0.08	0.51	0
9	0.91	0.22 (*SD* = 0.2)	0	0.44	0.46	0.54	0
10	0.83	0.22 (*SD* = 0.24)	1.90	0	−0.17	0.58	0
11	0.71	0.24 (*SD* = 0.24)	0	−0.51	0.44	0.34	0

**Table A2.** Participant-wise model results table from Experiment 1 with a proportional controller and gaze inputs.

	Performance			Trajectory correlation			
Participant	Yaw-rate correlation	Mean error (m)	Offtrack time (%)	WP 1	WP 2	WP 3	Excluded trials
1	0.69	0.26 (*SD* = 0.2)	0	0.62	0.42	−0.48	0
2	0.82	0.32 (*SD* = 0.29)	0	0.41	0.09	0.59	0
3	0.89	0.19 (*SD* = 0.19)	0	−0.06	0.38	0.86	0
4	0.87	0.26 (*SD* = 0.22)	0.01	0.14	0.18	0.25	1
5	0.90	0.11 (*SD* = 0.08)	0	0.63	0.55	0.74	0
6	0.82	0.14 (*SD* = 0.14)	0	0.42	0.49	0.45	0
7	0.86	0.17 (*SD* = 0.15)	0	−0.08	−0.44	0.49	0
8	0.87	0.15 (*SD* = 0.13)	0	−0.22	0.24	0.85	0
9	0.88	0.2 (*SD* = 0.18)	0	−0.02	0.53	0.31	0
10	0.81	0.2 (*SD* = 0.19)	0	−0.01	−0.21	0.22	0
11	0.72	0.26 (*SD* = 0.25)	0	−0.44	0.29	0.10	0

**Table A3.** Participant-wise model results table from Experiment 1 with a pure-pursuit controller and waypoint inputs.

	Performance			Trajectory correlation			
Participant	Yaw-rate correlation	Mean error (m)	Offtrack time (%)	WP 1	WP 2	WP 3	Excluded trials
1	0.81	0.19 (*SD* = 0.15)	0	0.01	0.23	0.69	0
2	0.88	0.27 (*SD* = 0.24)	0	0.80	0.10	−0.53	0
3	0.96	0.21 (*SD* = 0.26)	0	0.61	0.51	0.07	0
4	0.93	0.15 (*SD* = 0.14)	0	0.68	−0.01	−0.24	1
5	0.94	0.1 (*SD* = 0.09)	0	0.86	0.47	−0.34	0
6	0.92	0.12 (*SD* = 0.11)	0	0.94	0.17	0.30	0
7	0.93	0.12 (*SD* = 0.11)	0	0.26	−0.02	0.04	0
8	0.95	0.17 (*SD* = 0.14)	0	0.35	0.60	0.41	0
9	0.93	0.17 (*SD* = 0.16)	0	−0.07	0.74	−0.29	0
10	0.91	0.15 (*SD* = 0.14)	0	0.39	0.28	0.08	0
11	0.89	0.18 (*SD* = 0.22)	0	−0.12	0.02	0.10	0

**Table A4.** Participant-wise model results table from Experiment 1 with a proportional controller and waypoint inputs.

	Performance			Trajectory correlation			
Participant	Yaw-rate correlation	Mean error (m)	Offtrack time (%)	WP 1	WP 2	WP 3	Excluded trials
1	0.75	0.19 (*SD* = 0.15)	0	−0.74	0.81	0.71	0
2	0.85	0.27 (*SD* = 0.22)	0	0.79	0.07	−0.34	0
3	0.92	0.2 (*SD* = 0.23)	0	0.50	0.55	−0.35	0
4	0.90	0.15 (*SD* = 0.11)	0	0.48	0.03	0.18	1
5	0.91	0.12 (*SD* = 0.09)	0	0.89	0.25	0.71	0
6	0.91	0.11 (*SD* = 0.1)	0	0.40	−0.30	−0.41	0
7	0.88	0.11 (*SD* = 0.1)	0	0.56	−0.15	0.34	0
8	0.91	0.15 (*SD* = 0.12)	0	0.65	0.33	−0.33	0
9	0.89	0.16 (*SD* = 0.15)	0	−0.20	0.59	−0.26	0
10	0.87	0.14 (*SD* = 0.12)	0	0.12	−0.03	−0.34	0
11	0.83	0.19 (*SD* = 0.19)	0	−0.24	0.32	0.22	0

**Table A5.** Participant-wise position (referenced to the reference track/mean path) standard deviation of the true, human-driven trajectories in Experiment 1.

Participant	Position *SD* (m)	Offtrack time	Failed trials	Excluded trials
1	0.1797	0	0	0
2	0.2278	0	0	0
3	0.2012	0	0	0
4	0.1088	0	0	1
5	0.0902	0	0	0
6	0.1086	0	0	0
7	0.1012	0	0	0
8	0.1021	0	0	0
9	0.1347	0	0	0
10	0.1184	0	0	0
11	0.1736	0	0	0

**Table A6.** Participant-wise model results table from Experiment 2 with a pure-pursuit controller and gaze inputs.

	Performance			Trajectory correlation				
Participant	Yaw-rate correlation	Mean error (m)	Offtrack time (%)	Beginning	Middle	End	Failed trials	Excluded trials
1	0.98	0.55 (*SD* = 0.53)	3.54	0.38	0.14	−0.61	0	1
2	0.90	0.63 (*SD* = 0.29)	0.05	0.50	0.72	0.70	0	0
3	0.89	0.68 (*SD* = 0.95)	7.95	0.76	0.45	−0.99	1	2
4	0.98	0.38 (*SD* = 0.26)	0.85	−0.50	0.68	0.92	0	3
5	0.87	0.58 (*SD* = 0.38)	1.63	0.24	−0.20	−0.25	0	2
6								6
7	0.96	0.45 (*SD* = 0.29)	0.15	0.29	0.67	0.55	0	0
8	0.78	0.5 (*SD* = 0.42)	1.98	−0.41	0.13	0.28	0	0
9	0.98	0.44 (*SD* = 0.27)	0	0.10	0.44	0.03	0	0
10	0.98	0.22 (*SD* = 0.16)	0	−0.29	0.01	−0.30	0	0
11	0.94	0.58 (*SD* = 0.36)	0.83	0.20	−0.43	−0.71	0	0
12	0.96	0.42 (*SD* = 0.26)	0.21	0.17	0.25	−0.44	0	0
13	0.97	0.6 (*SD* = 0.3)	0	0.85	−0.52	0.64	0	0
14	0.98	0.47 (*SD* = 0.29)	0	0.65	0.70	0.10	0	0
15	0.92	0.43 (*SD* = 0.46)	2.29	0.38	−0.90	−0.01	0	0
16	0.97	0.76 (*SD* = 0.41)	2.93	−0.86	−0.86	−0.17	0	3

**Table A7.** Participant-wise model results table from Experiment 2 with a proportional controller and gaze inputs.

	Performance			Trajectory correlation				
Participant	Yaw-rate correlation	Mean error (m)	Offtrack time (%)	Beginning	Middle	End	Failed trials	Excluded trials
1	0.97	0.62 (*SD* = 0.44)	1.09	−0.89	0.06	−0.87	0	1
2	0.93	0.58 (*SD* = 0.31)	0.30	0.39	0.67	0.65	0	0
3	0.94	0.99 (*SD* = 0.67)	3.78	−0.90	0.86	−0.96	0	2
4	0.97	0.71 (*SD* = 0.38)	1.15	0.24	−0.95	−0.99	0	3
5	0.96	0.81 (*SD* = 0.42)	0	0.58	0.20	−0.36	0	2
6								6
7	0.96	0.64 (*SD* = 0.35)	0	0.52	0.34	0.07	0	0
8	0.93	0.54 (*SD* = 0.3)	0.04	0.21	0.45	−0.02	0	0
9	0.97	0.42 (*SD* = 0.28)	0.06	0.08	0.51	0.31	0	0
10	0.98	0.39 (*SD* = 0.2)	0	0.08	0.06	0.43	0	0
11	0.96	0.72 (*SD* = 0.4)	0	−0.67	−0.55	−0.73	0	0
12	0.97	0.48 (*SD* = 0.31)	0	−0.28	0.08	−0.42	0	0
13	0.96	0.78 (*SD* = 0.46)	0.22	0.82	−0.92	0.42	0	0
14	0.97	0.71 (*SD* = 0.34)	0	0.28	0.89	−0.22	0	0
15	0.97	0.6 (*SD* = 0.36)	0.02	−0.43	0.33	0.89	0	0
16	0.93	1.68 (*SD* = 0.78)	18.49	0.80	−0.18	0.96	0	3

**Table A8.** Participant-wise model results table from Experiment 2 with a pure-pursuit controller and waypoint inputs.

	Performance			Trajectory correlation				
Participant	Yaw-rate correlation	Mean error (m)	Offtrack time (%)	Beginning	Middle	End	Failed trials	Excluded trials
1	0.98	0.28 (*SD* = 0.2)	0	−0.49	0.26	−0.22	0	1
2	0.97	0.38 (*SD* = 0.22)	0	−0.53	−0.23	−0.28	0	0
3	0.98	0.51 (*SD* = 0.32)	0	−0.46	0.32	0.30	0	2
4	0.98	0.35 (*SD* = 0.23)	0	−0.58	0.60	−0.95	0	3
5	0.99	0.38 (*SD* = 0.17)	0	0.62	−0.42	−0.47	0	2
6								6
7	0.98	0.38 (*SD* = 0.24)	0	−0.43	0.19	0.52	0	0
8	0.98	0.31 (*SD* = 0.18)	0	−0.65	0.16	0.81	0	0
9	0.97	0.35 (*SD* = 0.28)	0	−0.61	−0.41	−0.53	0	0
10	0.99	0.23 (*SD* = 0.15)	0	0.14	0.43	−0.42	0	0
11	0.99	0.34 (*SD* = 0.22)	0	0.74	0.11	−0.44	0	0
12	0.98	0.3 (*SD* = 0.2)	0	−0.20	−0.18	0.01	0	0
13	0.98	0.51 (*SD* = 0.26)	0	0.66	−0.54	0.07	0	0
14	0.98	0.35 (*SD* = 0.22)	0	0.87	0.90	−0.79	0	0
15	0.98	0.28 (*SD* = 0.2)	0	−0.19	0.66	−0.12	0	0
16	0.97	0.64 (*SD* = 0.29)	0	0.26	−0.74	0.96	0	3

**Table A9.** Participant-wise model results table from Experiment 2 with a proportional controller and waypoint inputs.

	Performance			Trajectory correlation				
Participant	Yaw-rate correlation	Mean error (m)	Offtrack time (%)	Beginning	Middle	End	Failed trials	Excluded trials
1	0.92	0.19 (*SD* = 0.16)	0	−0.46	0.43	0.30	0	1
2	0.91	0.26 (*SD* = 0.18)	0	0.30	−0.51	−0.41	0	0
3	0.91	0.39 (*SD* = 0.28)	0	0.32	−0.44	−0.93	0	2
4	0.91	0.25 (*SD* = 0.19)	0	−0.89	−0.54	0.96	0	3
5	0.91	0.26 (*SD* = 0.15)	0	0.69	−0.41	0.30	0	2
6								6
7	0.91	0.27 (*SD* = 0.2)	0	−0.38	−0.14	−0.32	0	0
8	0.90	0.21 (*SD* = 0.14)	0	−0.46	−0.68	0.45	0	0
9	0.90	0.3 (*SD* = 0.24)	0	0.58	−0.60	0.46	0	0
10	0.91	0.16 (*SD* = 0.11)	0	0.17	−0.62	−0.03	0	0
11	0.91	0.24 (*SD* = 0.17)	0	0.42	0.21	−0.03	0	0
12	0.91	0.22 (*SD* = 0.16)	0	0.47	0.72	0	0	0
13	0.91	0.38 (*SD* = 0.22)	0	−0.08	−0.24	−0.78	0	0
14	0.91	0.24 (*SD* = 0.17)	0	0.90	0.11	−0.37	0	0
15	0.91	0.2 (*SD* = 0.15)	0	−0.41	0.76	0.59	0	0
16	0.91	0.5 (*SD* = 0.27)	0	−0.95	−0.99	−0.71	0	3

**Table A10.** Participant-wise lane position standard deviation of the true, human-driven trajectories in Experiment 2.

Participant	Position *SD* (m)	Offtrack time	Failed trials	Excluded trials
1	0.2694	0	0	1
2	0.2787	0	0	0
3	0.4533	0	0	2
4	0.3097	0	0	3
5	0.3162	0	0	2
6				6
7	0.3255	0	0	0
8	0.256	0	0	0
9	0.3887	0	0	0
10	0.204	0	0	0
11	0.2813	0	0	0
12	0.2896	0	0	0
13	0.4046	0	0	0
14	0.301	0	0	0
15	0.261	0	0	0
16	0.4947	0	0	3
