# Supplementary material for: Can gaze control steering?
Source: J Vis. 2023 Jul 21;23(7):12. doi: 10.1167/jov.23.7.12 (PMC10365140; doi:10.1167/jov.23.7.12)
Supplement: Supplement 1 [file jovi-23-7-12_s001.pdf]

Supplementary Information
van Gogh artwork used in the experiment

Year	Title
1888	Avenue at Arles with houses
1887	Edge of a wheat field with poppies
1889	Enclosed Field with Rising Sun
1888	Field with flowers near Arles
1890	Field with Poppies / Poppy field
1888	Flowering apricot orchard with trees / Apricot Trees in Blossom
1888	Flowering orchard, surrounded by cypress
1888	Garden at Arles / Flowering Garden with Path
1888	Green corn stalks
1890	Green Wheat Field
1890	House at Auvers
1883	In the Dunes
1889	Landscape from Saint-Rémy
1889	"Les Alpilles", mountain-landscape at Saint-Rémy
1889	Lilac Bush (Lilacs)
1890	Long Grass with Butterflies
1889	Mountain Landscape at Saint-Remy
1889	Olive Grove
1889	Olive Grove ('Olivenhain')
1889	Olive Grove ('Olive grove – Google Art Project')
1889	Olive Trees / Olive Grove
1889	Olive Trees / Olive Trees with yellow sky and sun
1889	Olive Trees with the Alpilles in the Background
1887	Park at Asnieres in Spring
1887	Path in the Woods
1888	Ploughed fields ('The furrows')
1888	Sunset at Montmajour
1889	The garden at the asylum at Saint-Rémy
1889	The Poplars at Saint-Rémy
1888	The Rocks / Rocks with Oak Tree
1888	The White Orchard
1887	Trees
1889	Undergrowth
1889	Undergrowth, with ivy
1889	View of the Church of Saint-Paul-de-Mausole
1890	Wheat fields with View of Auvers
1888	Wheatfield
1890	Wheatfield With Cornflowers
1888	Wheatfield with sheaves of grain
1888	Wheatfield ('Weizenfeld')

Source: commons.wikimedia.org
